# Venetoclax combination therapy induces deep AML remission with eradication of leukemic stem cells and remodeling of clonal haematopoiesis

**DOI:** 10.1038/s41408-021-00448-w

**Published:** 2021-03-19

**Authors:** Romain Vazquez, Claire Breal, Loria Zalmai, Chloe Friedrich, Carole Almire, Adrien Contejean, Sylvain Barreau, Eric Grignano, Lise Willems, Benedicte Deau-Fischer, Patricia Franchi, Marguerite Vignon, Justine Decroocq, Rudy Birsen, Lauriane Goldwirt, Sophie Kaltenbach, Lucile Couronne, Michaela Fontenay, Olivier Kosmider, Didier Bouscary, Nicolas Chapuis

**Affiliations:** 1 Assistance Publique-Hôpitaux de Paris, Centre-Université de Paris, Service d’Hématologie Biologique, Hôpital Cochin, Paris, France; 2 Assistance Publique-Hôpitaux de Paris, Centre-Université de Paris, Service d’Hématologie Clinique, Hôpital Cochin, Paris, France; 3 Université de Paris, Institut Cochin, CNRSUMR8104, INSERM U1016, A Member of OPALE Carnot Institute, The Organization for Partnerships in Leukemia, Paris, France; 4 Assistance Publique-Hôpitaux de Paris, Nord-Université de Paris, Laboratoire de Pharmacologie Biologique, UMRS976, Hôpital Saint Louis, Paris, France; 5 Assistance Publique-Hôpitaux de Paris, Centre-Université de Paris, Laboratoire d’Onco-Hématologie, Hôpital Necker-Enfants maladies, Paris, France; 6 Université de Paris, Institut Necker-Enfants Malades, INSERM U1151, Paris, France; 7 Université de Paris, Institut Imagine, INSERM U1163, Paris, France

**Keywords:** Acute myeloid leukaemia, Cancer stem cells

Dear Editor,

Treatment of acute myeloid leukemia (AML) in older patients remains challenging^1^. Promising results recently emerged in patients treated with the BCL-2 inhibitor venetoclax associated with low dose cytarabine (LDAC) or hypomethylating agents (HMAs)^2–4^. This clinical benefit may be due to leukemic stem cells (LSCs) eradication^5,6^. The association is well-tolerated with manageable toxicity but different mechanisms of resistance^7–10^ and heterogeneous responses in accordance with molecular patterns were observed^11^. We report here our single institution experience of venetoclax combination therapy for 19 consecutive patients with previously untreated AML ineligible to intensive chemotherapy. Clonal hematopoiesis and LSCs follow-up monitored by high throughput sequencing (HTS) and multiparametric flow cytometry (MFC) were analyzed.

Characteristics of patients are reported in Supplementary Tables 1 and 2. Their median age was 77 years and they often presented poor risk AML with molecular features detected in MDS and AML with myelodysplasia-related changes. Eleven patients (57.9%) had unfavorable cytogenetic risk. HTS (detailed in Supplementary information) identified a median number of four mutations (range, 1–8) in 24 different genes (Supplementary Tables 3 and 4). Fifteen patients (78.9%) had adverse risk ELN criteria^12^. MFC analysis (detailed in Supplementary information) identified leukemia-associated-immunophenotype (LAIP) in 11/17 patients (64.5%) and allowed a median LSCs quantification of 1.7% (range, 0.04–61) in 15/16 patients (93.7%) (Supplementary Fig. 1).

Twelve (63%) patients received a 400 mg daily dose of venetoclax whereas 4 (21%) and 3 (15.8%) patients were treated with a dose of 200 mg and 100 mg, due to concomitant posaconazole administration from the beginning of the treatment. For 11 patients (57.9%), the starting dose of venetoclax was decreased when posaconazole was introduced and/or in accordance with monitoring of the venetoclax plasma concentration. After dose adjustment, plasmatic concentration was always in line with expectations (Supplementary Fig. 2).

Hematological and gastrointestinal adverse events (AEs) were the most common toxicities. Gastrointestinal AEs were almost grade 1/2 but did not lead to venetoclax discontinuation. Most hematological AEs were grade 3/4: neutropenia (*n* = 13, 68.4%), anemia (*n* = 8, 42%), thrombocytopenia (*n* = 9, 47.4%), neutropenic fever (*n* = 12, 63%). Three bacterial pneumopathies occurred but no fungal infections. The 1- and 2-month mortality was of 0% and 5.3% (*n* = 1), respectively.

Sixteen out of 19 patients (84.2%) achieved complete remission (CR) or complete remission with incomplete blood recovery (CRi) with 12 patients (63.2%) achieving CR (Supplementary Table 5). For 10 of those patients (83.3%), CR was obtained after the first course of treatment. Interestingly, among patients with adverse cytogenetic risk (*n* = 11), the CR/CRi rate was also very good (9/11, 81.8%). Almost all patients with intermediate cytogenetic risk (7/8, 87.5%) obtained CR. The median duration of response (DOR) for all patients who achieved CR + CRi (*n* = 16) was 8.9 months (95% CI, 3.9-not reached (NR)) (Fig. 1a). However, the DOR for patients with adverse cytogenetic risk was significantly decreased [4.6 months, (95% CI, 3.7-NR) vs NR, (95% CI, 6.5-NR); *p* = 0.0433, Fig. 1a].

**Fig. 1:**
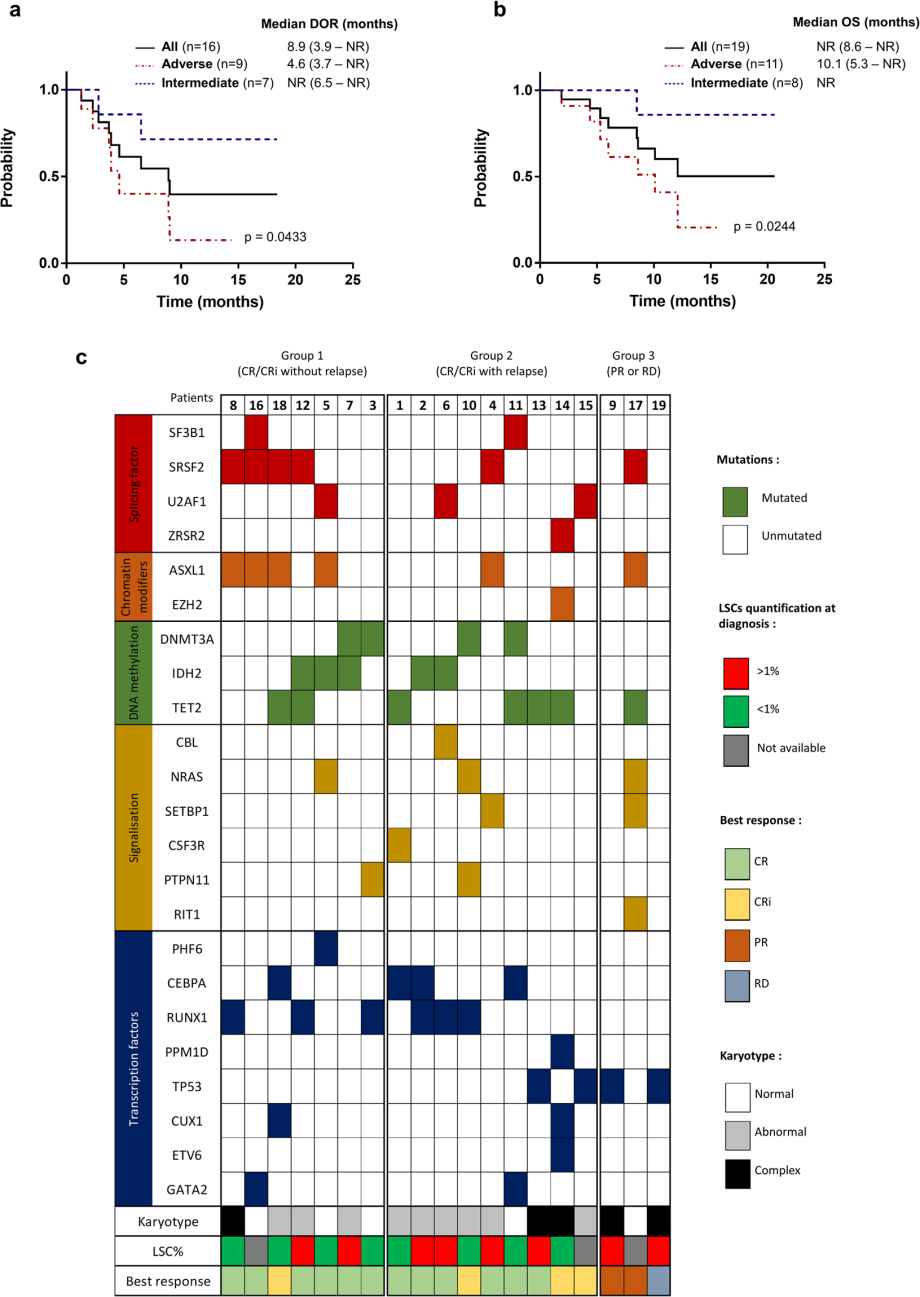
Outcome for patients treated by venetoclax combination therapy. **a** Median response duration for all patients who achieved CR/CRi and according to their adverse (red line) or intermediate (blue line) cytogenetic risk. **b** Overall survival for the entire patient cohort and according to their adverse (red line) or intermediate (blue line) cytogenetic risk. **c** Patients were categorized into those who achieved CR/CRi without relapse (group 1; *n* = 7) or with relapse at any time during the study (group 2; *n* = 9) and patients who only achieved partial remission (PR) or with a refractory disease (RD) (group 3; *n* = 3). The presence of study ID number, indicated mutations, complex karyotype, percentage of LSCs among the total blast population and best response are shown for each case.

Although well-tolerated, the association was difficult to maintain over a prolonged period, probably due to the fragility of the underlying haematopoiesis. Indeed, the median number of completed venetoclax cycles for patients achieving CR/CRi was 2,5 (range, 1–17). Venetoclax was discontinued for 11 patients (57.9%) after a median of 2 completed venetoclax cycles (range, 1–8) because of hematologic toxicity, leading to azaciditine monotherapy. Nine patients (56.3%) relapsed in a median time of 3.9 months (range, 1.3–8.9) and five of them were still treated at this time with venetoclax. Interestingly, the DOR of patients who stopped venetoclax was not significantly decreased [9 months (95% CI, 8.9-NR) vs 3.7 (95% CI, 2.3-NR); *p* = 0.0916, Supplementary Fig. 3], suggesting that early venetoclax discontinuation did not increase the relapse risk. In terms of overall survival (OS), the median follow-up was 10.7 months (range, 1.8–20.4) and the median OS for all patients was NR (95% CI, 8.6-NR) (Fig. 1b). The median OS for adverse AML cases was significantly decreased [10.1 months (95% CI, 5.3 -NR) vs NR; *p* = 0.0244, Fig. 1b]. Eight patients (42.1%) died in a median time of 7.2 months (range, 1.8–11.9) due to disease progression.

Patients were categorized into those who achieved CR/CRi without relapse (group 1; *n* = 7) or with relapse at any time during study (group 2; *n* = 9) and patients who only achieved partial remission (PR) or those with a refractory disease (RD) (group 3; *n* = 3) (Fig. 1c). Among patients of group 3, two had a TP53 mutation with a complex karyotype. The two other patients with TP53 mutations were from group 2. The OS period was significantly decreased for patients with TP53 mutations [6.5 months (95% CI, 1.9-NR) vs NR (95% CI, 10.1-NR); *p* = 0.0375, Supplementary Table 5]. Among patients of group 2, mutations leading to signaling pathways activation were more frequent compared to patients of group 1 (4/9, 44.4% vs 2/7, 28.6% respectively) and the DOR in these cases was decreased [6.75 months (95% CI, 3.9-NR) vs 9 months (95% CI, 3.7-NR)], although without achieving statistical significance. All patients carrying IDH2 mutations (*n* = 5) achieved CR and only 2/5 cases relapsed. The CR/CRi rate was increased in patients with a RUNX1 mutation but no association with prolonged response or increased OS was observed (Supplementary Table 5). SRSF2 mutations, strongly associated with ASXL1 mutations, were more frequently observed in group 1 and the DOR in these cases was increased [NR (95% CI, 8.9-NR) vs 4.6 months (95% CI, 3.7-NR)] but without achieving statistical significance (*p* = 0.09). Patients harboring a CEBPA mutation (*n* = 4) tended to have a shorter DOR than other cases [4.65 months (95% CI, 2.3-NR) vs 9 months (95% CI, 4.6-NR); *p* = 0.063]. An age >75 years and a high number of mutations at diagnosis (>4) did not affect the CR/CRi rate, the DOR or the OS (Supplementary Table 5). Finally, an LSCs level >1%, known to be associated with a poor prognosis^13,14^ was most frequent in group 3 (2/2) and group 2 (4/8, 50%) compared to group 1 (2/6, 33.3%) (Fig. 1c). Among patients with LSCs > 1%, 6/8 (75%) did not achieved CR/CRi or subsequently relapsed.

Minimal residual disease based on LAIP identification (LAIP-MRD) was quantified by MFC at each BM evaluation during follow-up (Supplementary Figs. 4 and 5). When patients achieved CR/CRi, the LAIP-MRD was negative in 9 of the 11 patients tested (81.8%) (Fig. 2a) attesting therefore to the depth of the response. Given that venetoclax specifically targets LSCs, we monitored LSCs of 14 patients during treatment. LSCs became undetectable during follow-up in 12 patients (85.7%) and all of them achieved CR/CRi (Fig. 2b). Disappearance of LSCs was observed from the first course of venetoclax combination therapy in eight patients. On therapy, LSCs-MRD and/or LAIP-MRD were evaluable for 13/16 patients who obtained a CR/CRi. For four of the six patients with durable response, the MRD remained always negative (Fig. 2c). In contrast, for 6/7 patients who relapsed, the treatment regimen led to an initially negative MRD that became detectable again during follow-up (Fig. 2d). Finally, the DOR for patients with at least one positive MRD assessment at any time during therapy (*n* = 9) was significantly shorter [4.6 months (95% CI, 3.7-NR) vs NR; *p* = 0.0378, Fig. 2e]. The OS was also decreased for these patients but without statistical significance (*p* = 0.2) (Supplementary Fig. 6). These results indicate therefore that venetoclax combination therapy induces deep responses and eradicates LSCs.

**Fig. 2:**
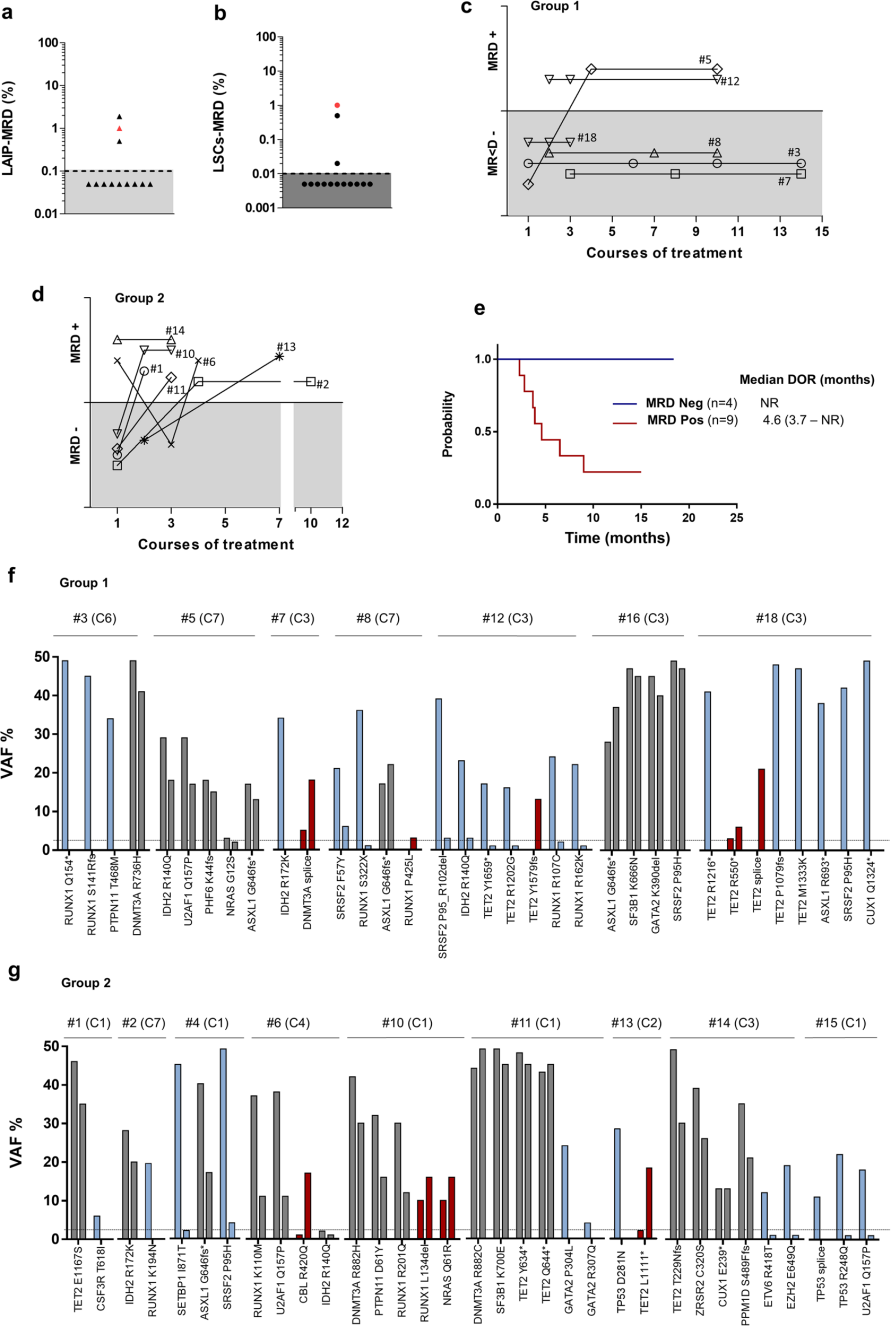
Monitoring of MRD and clonal haematopoiesis in BM of patients treated by venetoclax combination therapy. **a** Quantification of LAIP-MRD at first BM assessment after the beginning of treatment (after C1 (*n* = 9), after C2 (*n* = 2) or after C3 (*n* = 1)). Red points indicate patients who did not achieved CR/CRi. The Gray zone on the graph indicates the limit of quantification (0.1%). **b** Quantification of LSCs-MRD at first BM assessment after the beginning of treatment (after C1 (*n* = 10), after C2 (*n* = 3) or after C3 (*n* = 1)). Red points indicate patients who did not achieved CR/CRi. The Gray zone on the graph indicates the limit of quantification (0.01%). Follow up of MRD by MFC for patients who achieved CR/Cri without (group 1; *n* = 6) (**c**) or with relapse at any time during the study (group 2; *n* = 7) (**d**). A positive MRD was attested if either or both LAIP-MRD and/or a LSCs-MRD were detected above the limit of quantification. **e** Median response duration for all patients who achieved CR/CRi according their MRD status (either LAIP-MRD and/or LSCs-MRD) during the follow-up under treatment (always negative or at least one time positive). Follow-up of VAF mutations detected by HTS at different time points during therapy for patients who achieved CR/Cri without (group 1; *n* = 7) (**f**) or with relapse at any time during the study (group 2; *n* = 9) (**g**). Gray bars indicate mutations whose VAF only slightly decreased or increased during follow-up. Red bars indicates mutations whose VAF increased under therapy. Blue bars indicates mutations whose VAF strongly (at least 70% of decrease) decreased during treatment.

Clonal hematopoiesis was also investigated by HTS for all patients achieving CR/CRi. The mutation patterns detected at AML diagnosis were compared to the molecular features harbored by 6/7 patients with a prior MDS. For three patients who harbored at least one additional mutation at the time of MDS leukemic transformation, the combination therapy eradicated the sub-clonal population which raised at AML diagnosis and allowed obtaining CR/CRi (Supplementary Fig. 7a). For the three other patients, the molecular pattern did not change at AML diagnosis compared to the prior MDS (Supplementary Fig. 7b). Furthermore, we observed that 5/7 patients (71%) of group 1 experienced a strong decrease in most variant alleles frequencies (VAF) of the mutations detected at diagnosis (Fig. 2f). Interestingly, mutations for which the VAF only slightly decreased or even increased were DTA mutations which do not correlate with an increased relapse rate in AML patients treated with chemotherapy (Fig. 2f)^15^. In contrast, a strong decrease in most VAFs with a persistence or slight increase evident only in DTA mutations was observed for only three of the nine patients (33.3%) of group 2 (Fig. 2g). Furthermore, for these patients, the treatment clearly led to the selection of an initial sub-clonal population with IDH2, CBL or NRAS mutations (Fig. 2g). As expected, HTS performed at relapse in five patients confirmed the raise of the emergent mutations observed under treatment in two cases (IDH2 and CBL) (Supplementary Fig. 8). Thus, the type of mutations (DTA vs non-DTA) which persist or increase under treatment clearly affected patient’s outcomes.

Overall, our current study confirms the high efficacy and safety profile of the venetoclax combination therapy in older AML patients. The clinical benefits of this combination are associated with the efficient targeting of LSCs but we also show that a high amount of LSCs (>1%) at diagnosis may limit its efficacy. Finally, monitoring of MRD and clonal hematopoiesis in these patients seems useful approaches for predicting patient’s outcomes.

## Supplementary information

Supplementary Information
