# Supplementary material for: Venetoclax combination therapy induces deep AML remission with eradication of leukemic stem cells and remodeling of clonal haematopoiesis
Source: Blood Cancer J. 2021 Mar 19;11(3):62. doi: 10.1038/s41408-021-00448-w (PMC7979724; doi:10.1038/s41408-021-00448-w)
Supplement: Supplementary file 1 — Supplementary Information [file 41408_2021_448_MOESM1_ESM.doc]

**Supplementary Information for:**

**Venetoclax combination therapy induces deep AML remission with eradication of leukemic stem cells and remodeling of clonal haematopoiesis.**

Romain Vazquez, Claire Breal, Loria Zalmai, Chloe Friedrich, Carole Almire, Adrien Contejean, Sylvain Barreau, Eric Grignano, Lise Willems, Benedicte Deau-Fisher, Patricia Franchi, Marguerite Vignon, Justine Decroocq, Rudy Birsen, Lauriane Goldwirt, Sophie Kaltenbach, Lucile Couronne, Michaela Fontenay, Olivier Kosmider, Didier Bouscary, Nicolas Chapuis.

**SUPPLEMENTARY MATERIAL AND METHODS**

**Patients**

Since December 2018, each patient who had a diagnosis of AML according to the world health organization criteria in our institution (Hopitaux Universitaires Paris Centre) and who was not eligible to standard intensive chemotherapy due to the presence of various comorbidities (age older than 75 years, cardiac disease, prior anthracycline use or high probability of treatment-related mortality) were propose to receive treatment by venetoclax with azacitidine or LDAC. Patients had not received prior therapy for AML. Patients had an Eastern Cooperative Oncology Group (ECOG) PS 0-3. Key exclusion criteria were promyelocytic leukemia and active central nervous system involvement. This study was approved by local institutional review boards, and all patients provided written informed consent. The study was designed according to Good Clinical Practice Guidelines and the Declaration of Helsinki.

**Treatment and response assessment**

During cycle 1, patients were hospitalized to introduce the treatment if they had cytopenias. venetoclax (400mg/d) administrated orally once a day for 28 days was associated with azacitidine administrated subcutaneously days 1 to 7 (75mg/m^2^) (n=18) or with LDAC (20mg/m^2^x10 days) (n=1). Venetoclax plasma concentration was weekly determined using liquid chromatography with tandem mass spectrometric detection. The dose of venetoclax was reduced (200mg/d or 100mg/d) from the beginning or during cycle 1 in case of concomitant posaconazole administration for antifungal prophylaxis (if neutropenia <0,5 x 10^9^/L) and according to the plasma concentration level. All patients received supportive care measures if necessary (blood cell and platelets transfusions, antibiotics, antifungal prophylaxis). For all patients, a bone marrow (BM) evaluation was performed to evaluate blast clearance, after cycle 1 if they were hospitalized and later if they had ambulatory treatment. Response was assessed according the 2017 European Leukemia Network (ELN) criteria^1^. If there was persistent cytopenias at the end of cycle 1 without residual morphologic leukemia at the medullary evaluation, venetoclax was interrupted and granulocyte-colony-stimulating factors support was possible. The second cycle was delayed until the absolute neutrophil count recovery reached at least 0.5x10^9^/L. Subsequent cycles of venetoclax and azacitidine or LDAC were repeated as long time as there was no progression and no serious toxicity. Discontinuation and dose modification of venetoclax was possible at the discretion of the physician. Adverse events (AEs) were analyzed according to the National Cancer Institute Common Terminology Criteria for Adverse Events Version 4.0.

**Multiparametric flow cytometry**

Identification of LAIP (leukemia-associated-immunophenotype) and LSCs quantification were performed by MFC analysis on BM at AML diagnosis using the panel of antibodies listed below and the gating strategy described in Supplementary Fig. 1. BM specimens were collected into heparinized Hank’s medium (Eurobio, Courtaboeuf, France). BM purity was determined by the method of Holdrinet *et al* ^2^ and diluted samples (purity<50%) were excluded. Staining for MFC was performed within 24h following aspiration on 4 million BM cells after one wash of whole BM in Dulbecco’s phosphate buffered-saline (PBS, Eurobio). Cells were stained with antibodies described in supplemental Table 2. After 20 min incubation, red blood cells were lysed with 1 mL Versalyse^TM^ (Beckman Coulter) and the samples washed once in PBS. Cells were then resuspended in 500µL of PBS and data were acquired using a Navios flow cytometer (Beckman Coulter). At least 200,000 leucocytes were acquired for each patient to ensure the quality of the analysis. Data were analyzed using the Kaluza 2.1 version software (Beckman Coulter). Expression of CD19, CD7, CD56, abnormal pattern of CD13/CD33 or CD34/HLA-DR/CD117 expression were considered as LAIP if observed on at least 20% of leukemic cells. LSCs were quantified among the CD45+low leukemic bulk in the CD34+/CD38- fraction of cells by their expression of CD45Ra and/or CD123, CD7, CD56, CD19, CD97, TIM3, CLL1. For each BM assessment during the follow-up, similar MFC analysis was performed in order to quantify minimal residual disease (MRD) based on identification of LAIP (LAIP-MRD) and LSCs (LSCs-MRD) with a limit of quantification of 1x10^-3^ and 1x10^-4^, respectively ^3,4^.

| **Tube 1** | **Tube2** |
| --- | --- |
| **CD7 FITC** |  |
| A07755 (BC) |  |
| **CD56 PE** | **CD97/CLL1/TIM3** |
| A07788 (BC) | 555774/562566/563422 (BD) |
| **CD19 ECD** | **CD19** |
| A07770 (BC) | A07770 (BC) |
| **CD13 PC5.5** | **CD123** |
| B49196 (BC) | B20022 (BC) |
| **CD38 PC7** | **CD38 PC7** |
| 335825 (BD) | 335825 (BD) |
| **CD33 APC** |  |
| IM2471 (BC) |  |
| **CD34 AA700** | **CD34 AA700** |
| B92417 (BC) | B92417 (BC) |
| **CD117 AA750** | **CD45Ra APC-H7** |
| B92450 (BC) | 560674 (BD) |
| **HLA-DR PB** | **CD117 BV** |
| B36291 (BC) | 562434 (BD) |
| **CD45 KrOr** | **CD45 KrOr** |
| B36294 (BC) | B36294 (BC) |

**List of antibodies used for FCM analysis**

**Genomic testing**

Genomic DNA extracted from BM aspiration was studied by high throughput sequencing (HTS) of 45 genes recurrently mutated in myeloid malignancies (listed in Supplementary Table 3). We reported all clinically relevant variants with a VAF cut-off at 2% (pathogenic mutations detected are listed in Supplementary Table 4). Libraries were prepared using Ampliseq System, according to the manufacturer’s instructions, and sequenced on an ion torrent S5XL (Thermo Fisher Scientiﬁc, Inc., Waltham, MA, USA). Average coverage per gene was ≥ 600X. Reads were aligned against human genome build 19 (hg19) and analyzed for single nucleotid variant (SNV) calling with NextGENe software (SoftGenetics, Chicago, IL) and with an in-house pipeline (Polydiag, Institut Imagine, Université de Paris). Due to technical limitations, ASXL1 mutation hotspot (c.1934dupG) and mutational status of CEBPA and NPM1 were screened by a fragment analysis approach and by Sanger sequencing respectively.

**Statistics**

Data analysis was performed using GraphPadPrism 5.04 (GraphPad Software, La Jolla, CA). Duration of response (DOR) and Overall Survival (OS) were analyzed using Kaplan-Meier methodology. The study was closed to accrual on 1 August 2020.

**Supplementary Table 1: Patient and AML characteristics**

* Prior hematological disorder includes myelodysplastic syndromes (MDS) (n=7), chronic myelomonocytic leukemia (n=1), and multiple myeloma (n=1). One patient (#18) had an antecedent of AML with mutated NPM1 diagnosed in February 2010 and achieved a complete remission without minimal residual disease in February 2011 after autologous stem cell transplantation. The second AML he presented in February 2020 was negative for NPM1 mutation and was considered as a therapy related myeloid neoplasm. ELN, European LeukemiaNetWork; HMA, Hypomethylating agents; LSCs, Leukemic Stem Cells; WBC, White Blood Cells.

**Supplementary Table 2: Baseline information for each patient treated by venetoclax combination therapy.** CMML, Chronic Myelomonocytic Leukaemia; ELN, European LeukemiaNetWork; LSCs, Leukemic Stem Cells; MDS, Myelodysplastic Syndrome; MRC, Medical Research Council classification; WM, Waldenström Macroglobulinemia.

**Supplementary Table 3: List of the 45 genes sequenced by HTS**

**
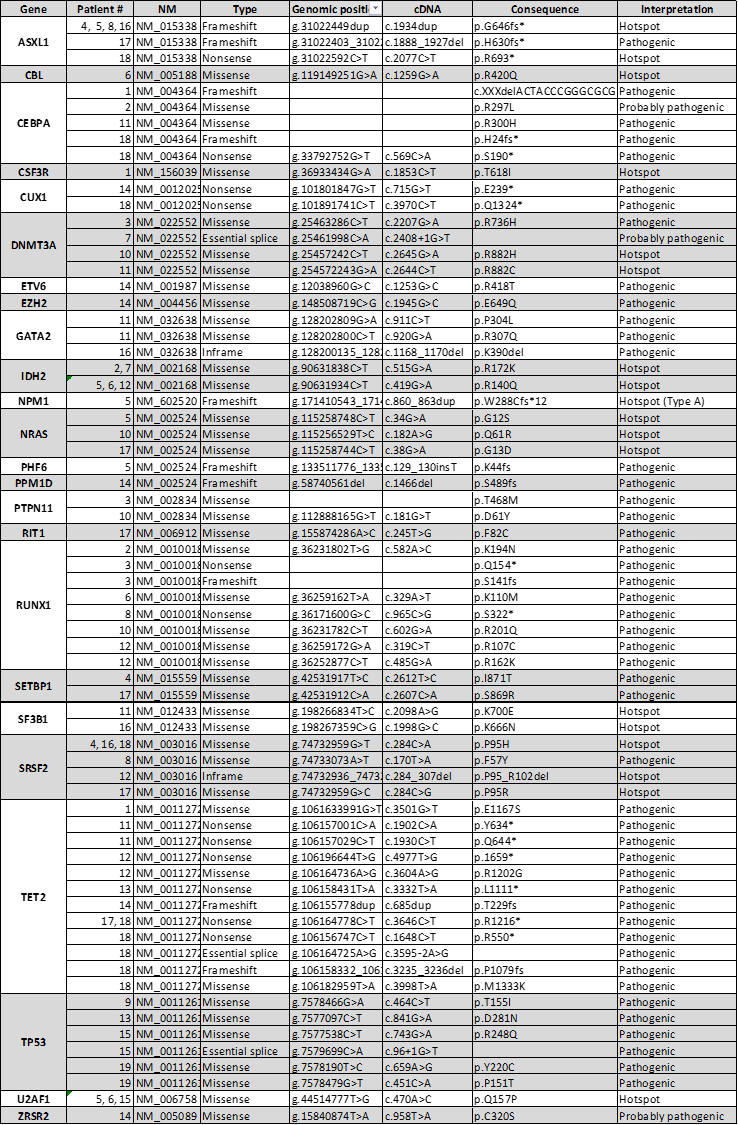
**

**Supplementary Table 4: List of pathogenic mutations detected by HTS**

**Supplementary Table 5: Response rates by subgroups of AML patients**

DOR, duration of response; LSCs, Leukemic Stem Cells; OS, overall survival.


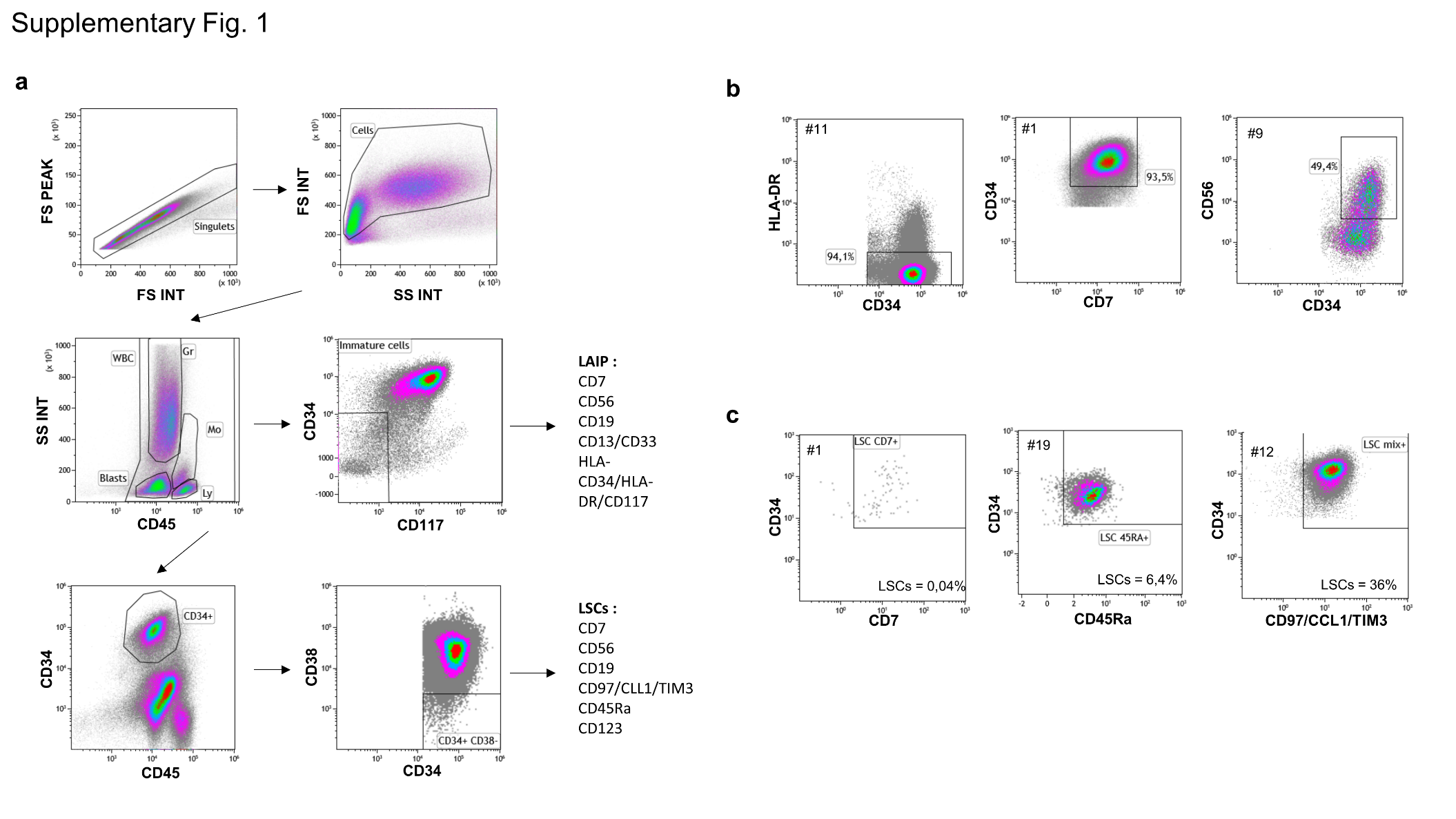


**Supplementary Fig. 1: Gating strategy for identification of LAIP and quantification of LSCs at diagnosis and follow-up of both LAIP-MRD and LSCs-MRD. a.** White blood cells (WBC) were selected as CD45^high^/SSC^int^ cells among living cells and singlets. Blasts cells were then gated according to their low CD45 expression and adjusted on a CD117/CD34 dotplot. Different dotplots were then analyzed to identify abnormal expression of CD7, CD19 and CD56 or abnormal pattern of CD13/CD33 or CD34/CDHLA-DR/CD117 expression (Tube 1). Different example of LAIP identified at diagnosis are shown in Figure 1A. For detection of LSCs, CD34+/CD38- cells among WBC were gated and different dot plots were then analyzed to identify abnormal expression of CD7 or CD56 (Tube 1) and CD45Ra, CD123, CD19 and CD97/CLL1/TIM3 (Tube 2). Cells with abnormal expression of at least one of these markers in Tube 1 or Tube 2 were then adjusted on a CD45/SSC dotplot. The same gating strategy was used for detection of both LAIP-MRD and LSCs-MRD. Identification at AML diagnosis of LAIP on CD45low cells at AML diagnosis **(b)** and of LSCs among CD34+/CD38- cells **(c).** Gr, Granulocytes; LAIP, Leukemia associated Immunophenotype; LSCs, Leukemic Stem Cells; Ly, Lymphocytes; Mo, Monocytes; WBC, White blood Cells.

**
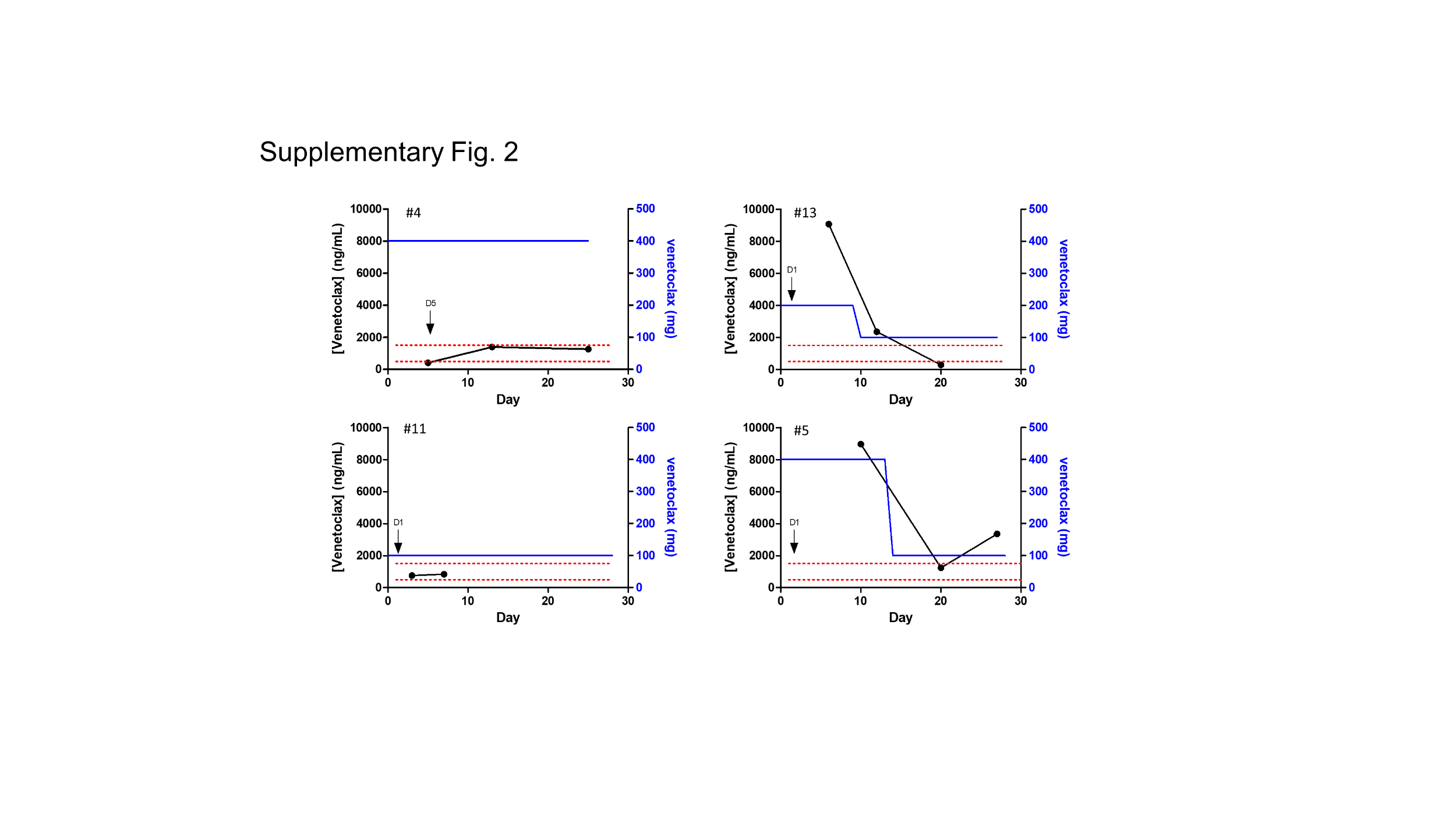
**

**Supplementary Fig. 2: Venetoclax dose adjustment and dosage of venetoclax plasmatic concentration during first course of treatment.**

The dark line indicates plasmatic venetoclax concentration (ng/mL). The blue line indicates the dose of venetoclax administrated to the patient. The red line indicate expected residual concentration of venetoclax (500-1500 ng/mL) ^5^. Arrow on each graph indicates the day on which the treatment by posaconazole was introduced.

**
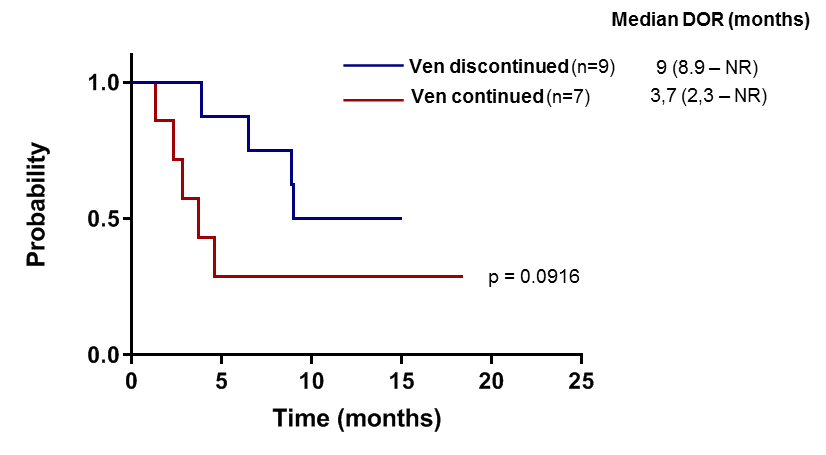
**

**Supplementary Fig. 3: Venetoclax discontinuation does not alter the DOR of venetoclax combination therapy.**

Median response duration for patients with (blue line) or without (red line) venetoclax discontinuation during the follow-up.

**
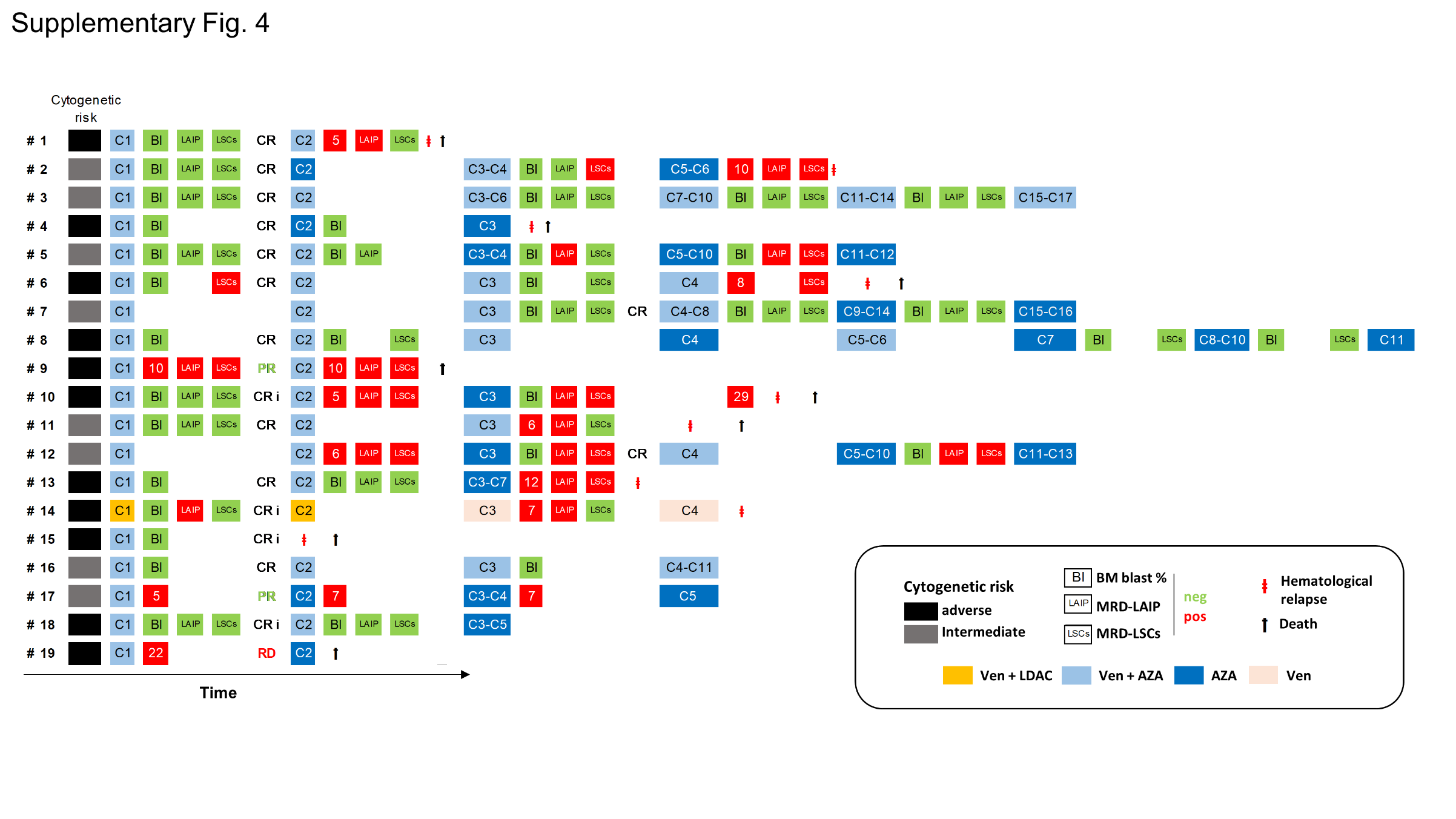
**

**Supplementary Fig. 4: Follow up of all patients treated with venetoclax combination therapy.**

Study ID number, best response, monitoring of bone marrow blast cells and MRD based on LAIP and/or LSCs, adaptation of venetoclax combination therapy and outcome (death or haematological relapse) are indicated for each patients at different time point during the treatment.

**
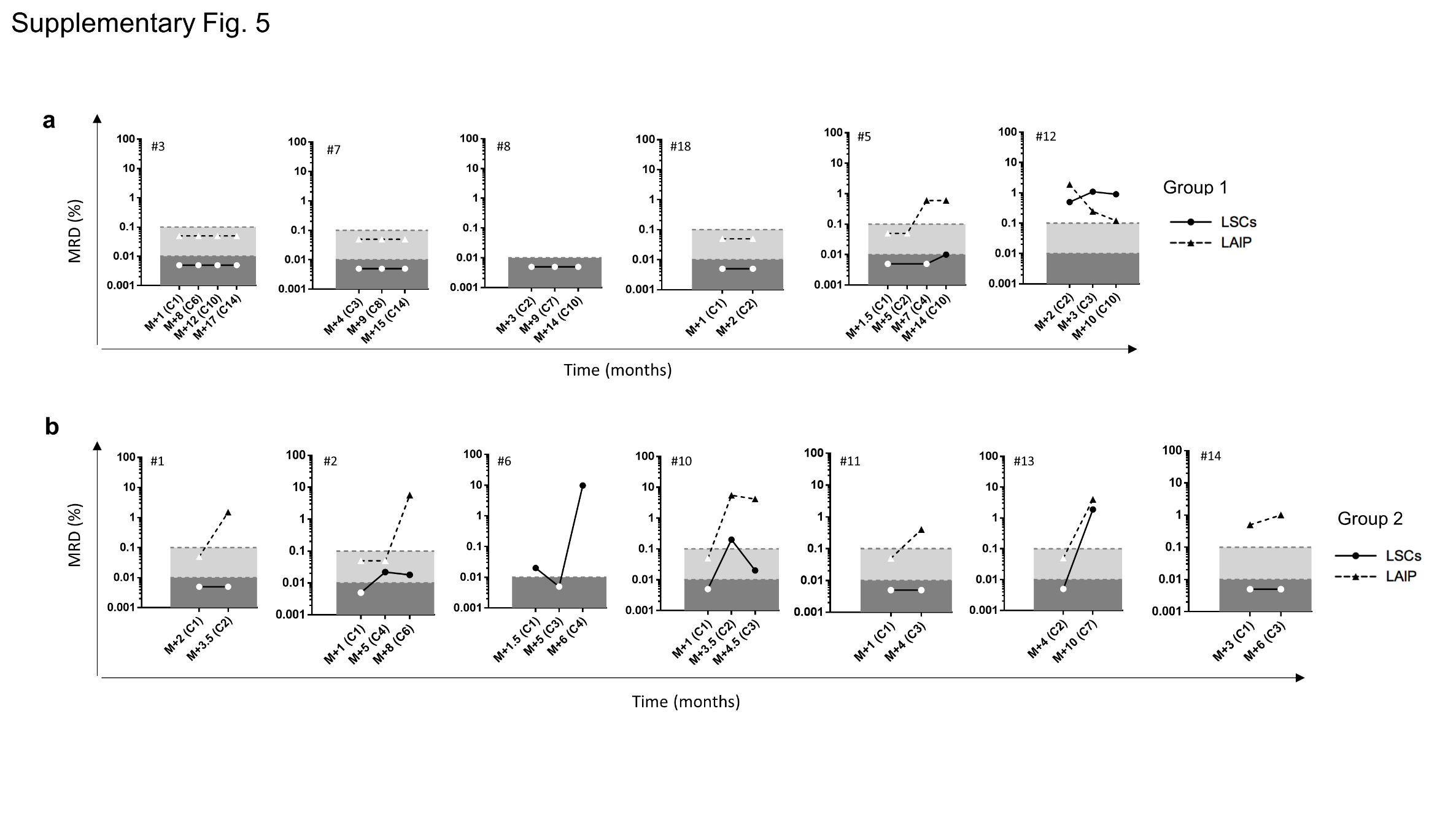
**

**Supplementary Fig. 5: follow up of LAIP-MRD and LSCs-MRD during treatment by FCM**.

Patients were categorized into those who achieved CR/CRi without relapse (group 1; n=6) **(a)** or with relapse at any time during the study (group 2; n=7) **(b)**. Black points indicate positive detection of MRD and white points indicate MRD undetecatable according the limit of detection (0.1% for LAIP-MRD and 0.01% for LSCs-MRD). Time in months after AML diagnosis and the number of courses of treatment is indicated on the X axis.

**
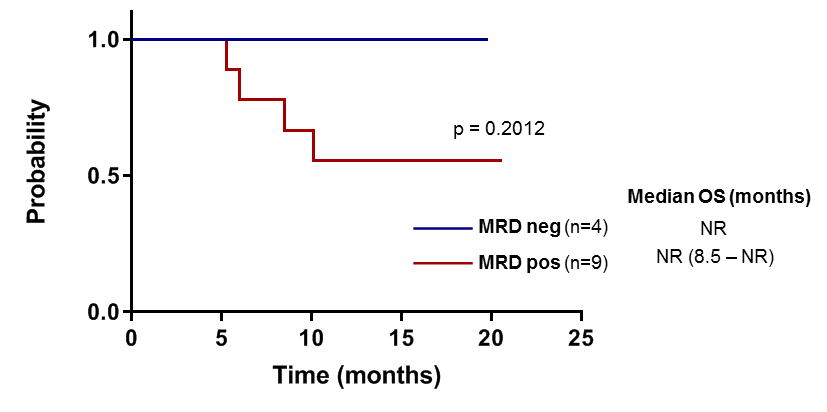
**

**Supplementary Fig. 6: Median OS for all patients who achieved CR/CRi according their MRD status (either LAIP-MRD and/or LSCs-MRD) during the follow-up under treatment** (always negative or at least one time positive).

**
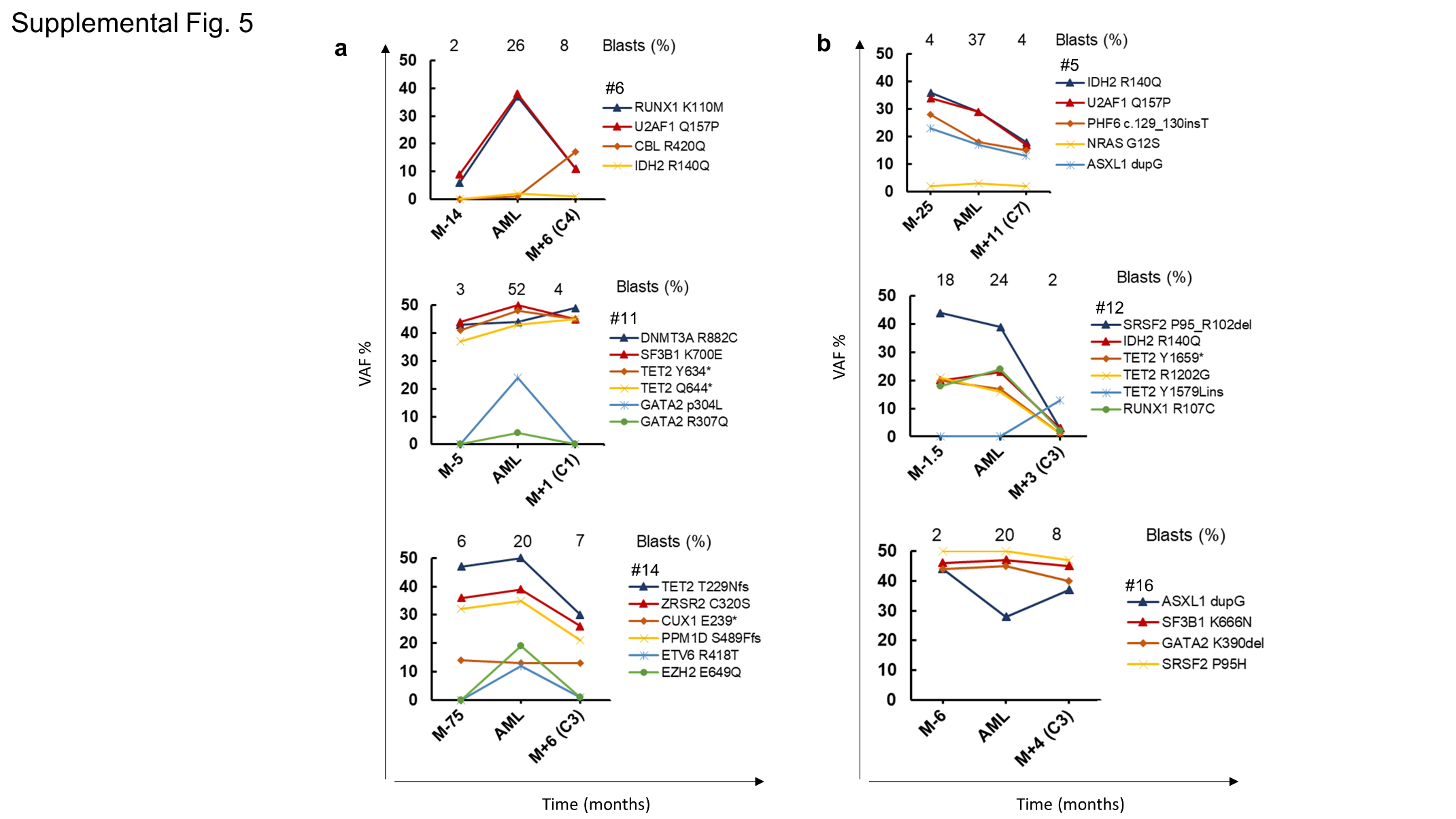
**

**Supplementary Fig. 7: follow up of clonal hematopoiesis for patients with prior SMD treated by venetoclax combination therapy.**

Impact of venetoclax combination therapy on VAF for patients with **(a)** or without expansion of a subclonal population at AML diagnosis **(b).** The percentage of BM blast cells on each samples is indicated on the top of each graph. Time in months before and after AML diagnosis and the number of courses of treatment is indicated on the X axis.

**
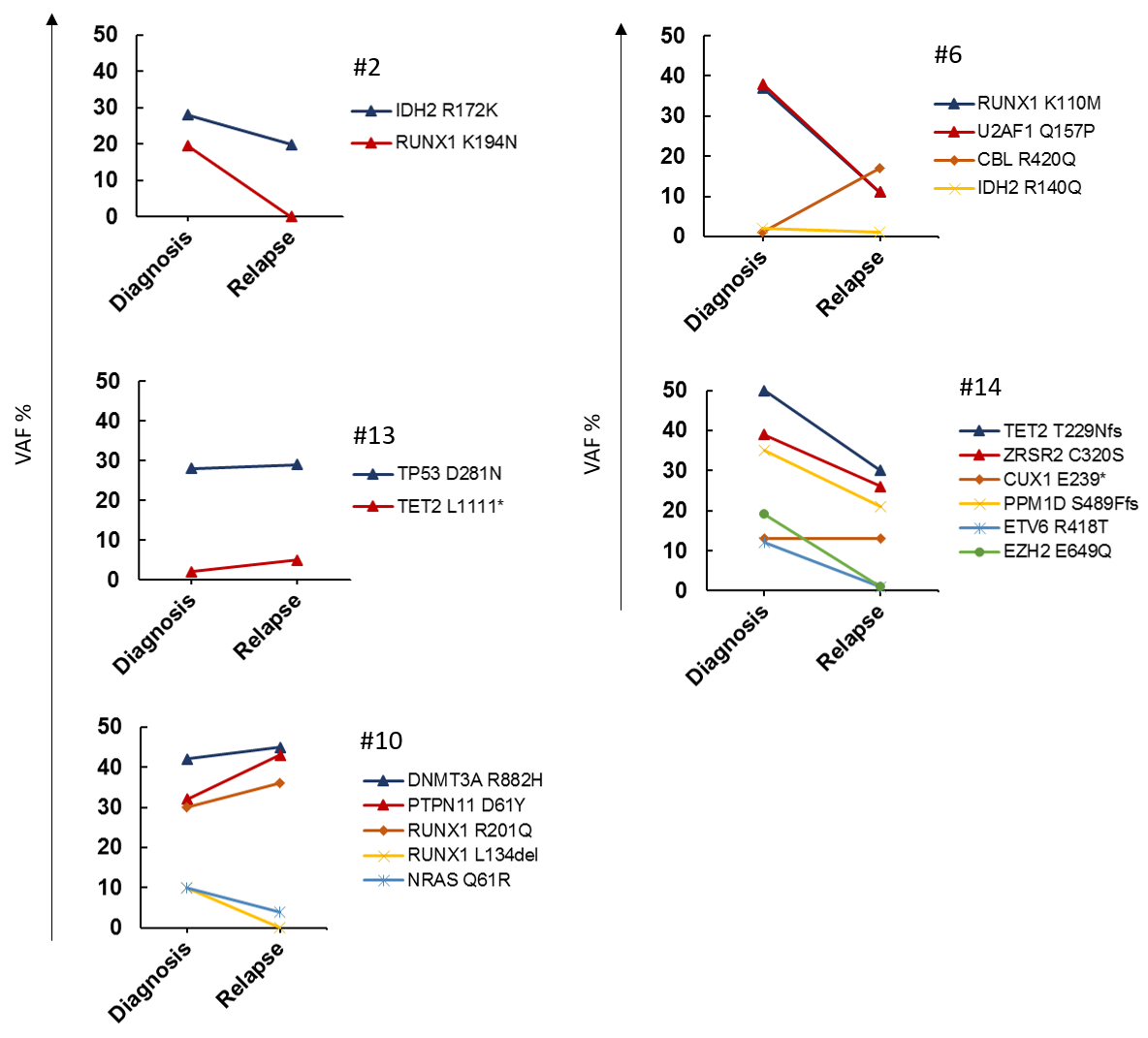
**

**Supplementary Fig. 8: Pairwise comparison of mutation status and VAF in AML patient samples at diagnosis and at relapse under treatment.**

**REFERENCES**

1 Döhner H, Estey E, Grimwade D, Amadori S, Appelbaum FR, Büchner T *et al.* Diagnosis and management of AML in adults: 2017 ELN recommendations from an international expert panel. *Blood* 2017; **129**: 424–447.

2 Holdrinet RS, von Egmond J, Wessels JM, Haanen C. A method for quantification of peripheral blood admixture in bone marrow aspirates. *Exp Hematol* 1980; **8**: 103–107.

3 Schuurhuis GJ, Heuser M, Freeman S, Béné M-C, Buccisano F, Cloos J *et al.* Minimal/measurable residual disease in AML: a consensus document from the European LeukemiaNet MRD Working Party. *Blood* 2018; **131**: 1275–1291.

4 Freeman SD, Hourigan CS. MRD evaluation of AML in clinical practice: are we there yet? *Hematology* 2019; **2019**: 557–569.

5 Salem AH, Agarwal SK, Dunbar M, Nuthalapati S, Chien D, Freise KJ *et al.* Effect of Low- and High-Fat Meals on the Pharmacokinetics of Venetoclax, a Selective First-in-Class BCL-2 Inhibitor. *J Clin Pharmacol* 2016; **56**: 1355–1361.
